# An Interesting Fistula Tract Presenting with Recurrent Gluteal Abscess: Instructive Case

**DOI:** 10.1155/2015/682842

**Published:** 2015-04-05

**Authors:** Gulsum Iclal Bayhan, Ozge Metin, Burak Ardicli, Ayse Karaman, Gonul Tanir

**Affiliations:** ^1^Department of Pediatric Infectious Disease, Yuzuncu Yil University, 65000 Van, Turkey; ^2^Department of Pediatric Infectious Disease, Dr. Sami Ulus Maternity and Children's Training and Research Hospital, 06080 Ankara, Turkey; ^3^Department of Pediatric Surgery, Dr. Sami Ulus Maternity and Children's Training and Research Hospital, 06080 Ankara, Turkey

## Abstract

A fistula extending from the gluteus to penis is an extremely rare entity. In this paper, we have highlighted novel variant of congenital penile to gluteal fistula complicated with gluteal and penoscrotal abscess in a previously healthy boy. A fistulous tract extending from the gluteus to penis has been shown by fistulogram. Bleomycin has been used in fistula tract with successful results in our patient.

## 1. Introduction

A fistula extending from the gluteus to penis is an extremely rare entity. We report a patient with recurrent gluteal abscess having an unusual gluteal-penile fistula along the posteroanterior body axis.

## 2. Case

A 4.5-year-old boy presented to our hospital with complaint of recurrent abscess on the right hip since he was 7 months old. He had been diagnosed as having gluteal abscess accompanied with penile abscess previously on seven separate occasions and incision and drainage were performed. He was operated on for hypospadias by using Snodgrass technique at two years old. A month later, recurrent abscesses in the gluteal region had been excised; pathology reported chronic inflammation and congestion in fibroadipose tissue. During our hospital admission, abscesses on penile and gluteal sides were detected at the same time. Physical examination revealed discharging sinus surrounded with 2 × 2 cm induration on the right gluteus and discharging sinus on the penoscrotal junction. Abscess tract was palpated through to perineal region. Anorectum was normal.

**Figure 1:**
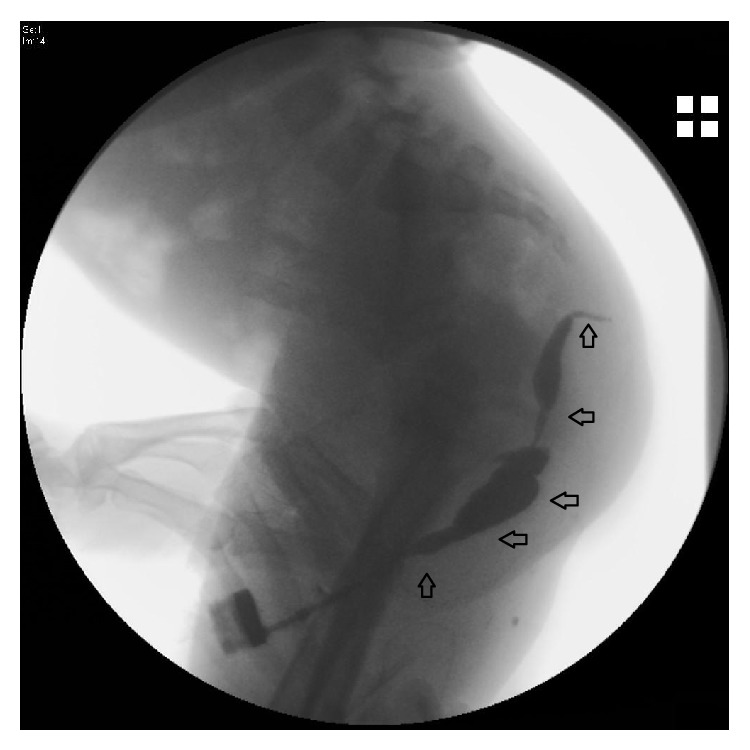
Fistulography of the patient. The opaque material was given from the mouth of the fistula at the level of the penoscrotal region. The opaque material extended superiorly to the inferior level of the coccyx and terminated under the skin.

Laboratory findings of the patient were as follows: white cell count was 11.9 × 10^3^/*μ*L (normal range, 5.1–15.5 × 10^3^/*μ*L), hemoglobin 9.5 g/dL, platelet count 460 × 10^3^/*μ*L, and C-reactive protein 32 mg/L (normal range, 0–8 mg/L). He was hospitalized and ceftriaxone was commenced after pus culture was obtained from both sites. Ultrasound examination revealed 31 × 22 mm lobulated contoured abscess forming dense content with the acoustic empowerment in the right gluteal region which reached to intergluteal region. Penoscrotal USG revealed 5 × 4 mm sized dense content abscess within the corpus cavernosum in the proximal part of the ventral penile adjacent to the left lateral side of the urethra.* Enterococcus* spp. had grown on his both pus cultures. Antibiotic treatment was switched to teicoplanin. The swellings in the gluteus and penoscrotal junction were gradually decreased in size with antibiotic treatment. A fistulography was performed and showed a long sinus tract extending from the region of the penoscrotal junction to the right gluteal region (Figure 1). There was no connection with the urinary and gastrointestinal tract.

Surgical treatment was required to be an extensive procedure with great risk of developing intraoperative damage to vital organs and nerves. Instead, sclerotherapy was administered to induce healing of the lesion. Penoscrotal sinus was catheterized and diluted bleomycin at a dose of 0.25 mg/kg was administered under general anesthesia. Postoperative recovery was uneventful, and the patient was discharged after 3 days. Six months later, the skin healed completely. The patient has been followed for one and half years and abscess was not repeated until now.

## 3. Discussion

The gluteal abscesses and fistula have been reported as complications of Crohn's disease, diverticulitis, colon carcinoma, and tuberculosis of lumbar vertebrae [1–3]. In our patient, recurrent abscesses history in both penoscrotal region and gluteus and the cultures from both abscess sides yielding the same microorganism suggested the possibility that there had been a fistula. In the case presented, initial diagnosis of a fistula could not be made till the fistulography was conducted. Despite the newer imaging modalities, a fistulography is still the best means of evaluating a sinus tract or fistula when an external communication is present. The fistulography allows visualization of the fistula tract and origin. Computed tomography is helpful if exact spatial delineation of the tract is necessary or a suspect of associated abscess exists. Because CT results in high radiation, it should be used carefully in young children in selected indications. Ultrasound examination is generally not useful since it is limited by bowel gas and surgical incisions [4, 5]. Magnetic resonance imaging (MRI) is reported as the golden standard in preoperative assessing and classifying of fistula, because MRI allows direct visualization of the tracts and abscesses through to high soft tissue resolution [6, 7].

Surgery has been the main therapy for any sinus and fistula tracts. Open surgical exploration and repair provide definitive management, avoid recurrence, and prevent infection. Although surgical excision has been considered as a mode of treatment by most of the surgeons, the patient may be faced with some conditions such as nerve injuries, prolonged lymphatic drainage from the wound, recurrent lesions, wound infections, and unacceptable scar formations. Sclerotherapy using bleomycin is an established technique for the treatment of developmental vascular anomalies, also those which are at risk of developing intraoperative damage to vital organs and nerves, and lymphangiomas. Now sclerotherapy has been successfully used in the treatment of congenital sinus tracts [8–10]. Bleomycin is an antitumor agent and, besides its antineoplastic effect, bleomycin causes nonspecific inflammatory reaction leading to fibrosis in the surrounding tissues [10]. Bleomycin has been used in fistula tract with successful results in our patient. No procedure-related complications and no recurrence were observed in our patient.

In conclusion, the present case is a description of a novel fistula tractus. To the best of our knowledge, this is the first case with fistula deeply located and extending from the gluteus to penis in the literature. Clinicians should consider underlying congenital malformation in the differential diagnosis of recurrent perineal and/or gluteal abscess. Catheter-based bleomycin injection could be applied as a safe, minimally invasive, and effective option for complex gluteal fistula, which makes it a suitable and durable alternative to open surgery. Treatment of this entity is individualized according to the site of fistula and associated anomalies, as well as the condition of the distal urethra.
